# Integrated analysis of single-cell and Bulk RNA sequencing reveals a malignancy-related signature in lung adenocarcinoma

**DOI:** 10.3389/fonc.2023.1198746

**Published:** 2023-06-23

**Authors:** Mengxi Wu, Zhenyu Wu, Jun Yan, Jie Zeng, Jun Kuang, Chenghua Zhong, Xiaojia Zhu, Yijun Mo, Quanwei Guo, Dongfang Li, Jianfeng Tan, Tao Zhang, Jianhua Zhang

**Affiliations:** ^1^ Department of Thoracic Surgery, Shenzhen Hospital, Southern Medical University, Shenzhen, China; ^2^ Department of Urology, The First People’s Hospital of Foshan, Foshan, China; ^3^ Department of Thoracic Surgery, Guangzhou First People’s Hospital, South China University of Technology, Guangzhou, China

**Keywords:** lung adenocarcinoma, ScRNA-seq, malignancy, gene signature, prognosis

## Abstract

**Background:**

Lung adenocarcinoma (LUAD), the most common histotype of lung cancer, may have variable prognosis due to molecular variations. The research strived to establish a prognostic model based on malignancy-related risk score (MRRS) in LUAD.

**Methods:**

We applied the single-cell RNA sequencing (scRNA-seq) data from Tumor Immune Single Cell Hub database to recognize malignancy-related geneset. Meanwhile, we extracted RNA-seq data from The Cancer Genome Atlas database. The GSE68465 and GSE72094 datasets from the Gene Expression Omnibus database were downloaded to validate the prognostic signature. Random survival forest analysis screened MRRS with prognostic significance. Multivariate Cox analysis was leveraged to establish the MRRS. Furthermore, the biological functions, gene mutations, and immune landscape were investigated to uncover the underlying mechanisms of the malignancy-related signature. In addition, we used qRT-PCR to explore the expression profile of MRRS-constructed genes in LUAD cells.

**Results:**

The scRNA-seq analysis revealed the markers genes of malignant celltype. The MRRS composed of 7 malignancy-related genes was constructed for each patient, which was shown to be an independent prognostic factor. The results of the GSE68465 and GSE72094 datasets validated MRRS’s prognostic value. Further analysis demonstrated that MRRS was involved in oncogenic pathways, genetic mutations, and immune functions. Moreover, the results of qRT-PCR were consistent with bioinformatics analysis.

**Conclusion:**

Our research recognized a novel malignancy-related signature for predicting the prognosis of LUAD patients and highlighted a promising prognostic and treatment marker for LUAD patients.

## 1 Introduction

Lung cancer is a malignant tumor with the highest mortality rate in the world (1). Lung adenocarcinoma (LUAD) is the most common histotype of lung cancer, accounting for around 40% of all lung malignancies with an increasing prevalence (2). Despite the employment of novel therapies including targeted therapy and immunotherapy, the prognosis for patients with LUAD was dismal (3). Although patients may have comparable pathology, anatomical location, and clinical staging, their survival outcomes will likely vary because of molecular differences. Therefore, it is necessary to exploit newly prognostic molecular biomarkers, which will be of assistance in improving the prognosis of overall survival (OS) as well as the therapy effectiveness for LUAD patients.

In recent years, next-generation sequencing has been widely used, but conventional NGS does not detect cellular heterogeneity (4–6). Single-cell RNA sequencing (scRNA-seq) can be used to detect the genome, transcriptome, and other multi-omics of single cells. It is a powerful approach to dissect cellular heterogeneity, which can specifically obverse the changes in the tumor microenvironment (TME) (7). It is known that tumor cells are surrounded by TME, including a variety of immune cells, stromal cells, extracellular matrix molecules, and various cytokines (8). As a key priority, tumor cells play a vital role in the occurrence and development of tumors. In this study, we extracted tumor cell subpopulations and identified tumor cell marker genes through scRNA-seq.

In the research, we applied the scRNA-seq data from Tumor Immune Single Cell Hub (TISCH) database to obtain a gene expression map from the level of single cells in LUAD. Next, we extracted transcriptome data and associated clinical information from TCGA database (TCGA-LUAD) and Gene Expression Omnibus (GEO) database. Subsequently, we used TCGA cohort as the training set while the GSE68465 and GSE72094 cohorts as the validation sets. By linking associated genes with clinical cases of LUAD, we focused on investigating the effect of the malignancy-related signature on foretelling the mortality of LUAD patients and exploring their underlying mechanisms on tumor growth and progression.

## 2 Materials and methods

### 2.1 Dataset source and preprocessing

First, we downloaded the single cell RNA sequencing (scRNA-seq) dataset (GSE117570) (9) of 4 LUAD patients from the TISCH database^1^. RNA-sequencing (RNA-seq) and the matched clinical characteristics of 460 LUAD patients were derived from the TCGA database^2^. We downloaded two datasets (439 LUAD patients for GSE68465 and 386 patients for GSE72094) of RNA expression data and complete clinical data from the GEO database^3^ (10, 11). The batch effect was adjusted through the “sva” R package. The IMvigor210 cohort (12) of bladder cancer patients treated with anti-PD-L1 therapy was obtained through the “IMvigor210CoreBiologies” R package, and the GSE91061 dataset (13) that accepted anti-PD-1 and anti-CTLA4 treatment was also attained to predict the efficiency of immunotherapy. The basic information of series was shown in **Supplementary Table 1**.

### 2.2 ScRNA-seq analysis

Quality control and normalization of scRNA-seq data were processed with the “Seurat” R package. Cell clusters were derived from the TISCH database. According to the documentation, the cell type was annotated by the description provided by the original study, the markers of malignant cells, and the “inferCNV” R package. FindAllMarkers function was used to determine and annotate gene markers for different cell clusters with thresholds of *p*.adjust< 0.05 and log2 [Foldchange] > 0.3. We extracted the marker genes of the malignant celltype for further study.

### 2.3 Generation of malignancy-related signature

To establish the malignancy-related signature, we employed TCGA cohort as the training set, while the GSE68465 and GSE72094 datasets were the validation sets. Univariate Cox analysis was performed to explore prognosis-associated genes (*p<* 0.001). Random survival forest (RSF) analysis was then conducted using the “randomForestSRC” R package to further narrow down the prognostic gene panel. In RSF analysis, variables were ranked by minimal depth, of which a smaller value indicated greater predictiveness. Next, multivariate Cox regression analysis was used to establish the optimal malignancy-related signature based on respective coefficients (β) and gene expression levels (Exp). This formula was used to calculate each patient’s malignancy-related risk score (MRRS). Subsequently, we divided the patients into high- and low-risk groups based on the median MRRS. The Kaplan-Meier approach was applied to determine the prognostic difference between the two groups. We further evaluate the correlations between the MRRS and clinical features including age, gender, clinical stage, TN stages, and smoking status. Univariate and multivariate Cox analyses were utilized to assess the prognostic significance of MRRS. Meanwhile, we collected the GSE68465 and GSE72094 cohorts to verify MRRS’s predictive efficacy.

### 2.4 Functional enrichment analysis

To investigate the underlying mechanism regarding MRRS, differentially expressed genes were obtained between the high- and low-risk cohorts. First, we performed Gene Ontology (GO) enrichment and Kyoto Encyclopedia of Genes and Genomes (KEGG) pathway analyses using the “clusterProfiler” R package. GO and KEGG terms with *p*< 0.05 were visualized by the “circlize” R package. GSVA was employed to determine the differences between the two cohorts on the oncogenic hallmark pathways (h.all.v7.1.symbols) deposited in the MSigDB database (*p*.adjust< 0.001). Gene Set Enrichment Analysis (GSEA) was performed between the two groups for the same hallmark pathways with the “GSEA” R package (FDR< 0.25, NES > 1, and *p*.adjust< 0.05). Kaplan-Meier method was employed to determine the prognostic significance of the overlapping hallmark pathways of GSVA and GSEA.

### 2.5 Somatic mutation analysis

The somatic mutations of LUAD patients were extracted from TCGA database. The “maftools” R package explored the specific somatic mutation variations in different MRRS groups. Next, we investigated the mutually co-occurring or exclusive mutations, tumor-causing genes, and enrichment of known oncogenic pathways between the two cohorts. Tumor mutation burden (TMB) reflecting total mutation counts for each LUAD patient was computed and tested for correlation with MRRS. In addition, we analyzed the predictive value of TMB and MRRS on the survival outcomes in terms of the MRRS risk cohorts.

### 2.6 Immune landscape analysis and treatment response prediction

We compared the high- and low-risk groups’ immune cell abundance, immune function, and immune checkpoints. The Tumor Immune Dysfunction and Exclusion (TIDE) algorithm^4^ (14) was applied to predict the potential immunotherapy response based on the RNA expression profile of LUAD patients. The IMvigor210 and GSE91061 datasets were also used to determine the correlation between the MRRS and potential immunotherapy efficacy. Meanwhile, we investigated the chemotherapy response of the two groups, and the “oncoPredict” R package predicted the therapeutic effect of chemotherapeutic drugs for each patient.

### 2.7 RNA extraction and quantitative real-time PCR

Human LUAD cells (A549, H1299, SKLU1, and H1250) and normal bronchial epithelial cells (16HBE) were obtained from Procell Life Science and Technology (Wuhan, China). Total RNA of the cells was isolated using TRIzol reagent (BioTeke, Beijing, China). Subsequently, qRT-PCR was performed utilizing the HiScript II Q RT SuperMix for qPCR (Bioer Technology, Hangzhou, China). The 2−△△Ct method was used to calculate the relative expression of each lnRNA. The differences in the expression levels of LUAD cells and normal cells were assessed using the unpaired t test. The primer sequences were showed in **Supplementary Table 2**.

### 2.8 Statistical analysis

We conducted all statistical analyses in this research using R software (version 4.2.2). All codes of the analysis was uploaded in GitHub^5^. The unpaired Student’s t-test was used to assess the differences between continuous variables. The chi-square test was utilized to examine the relationship between categorical factors. Statistical significance for most analyses was empirically set at a two-tailed *p*< 0.05.

## 3 Results

### 3.1 ScRNA-seq analysis

**Figure 1:**
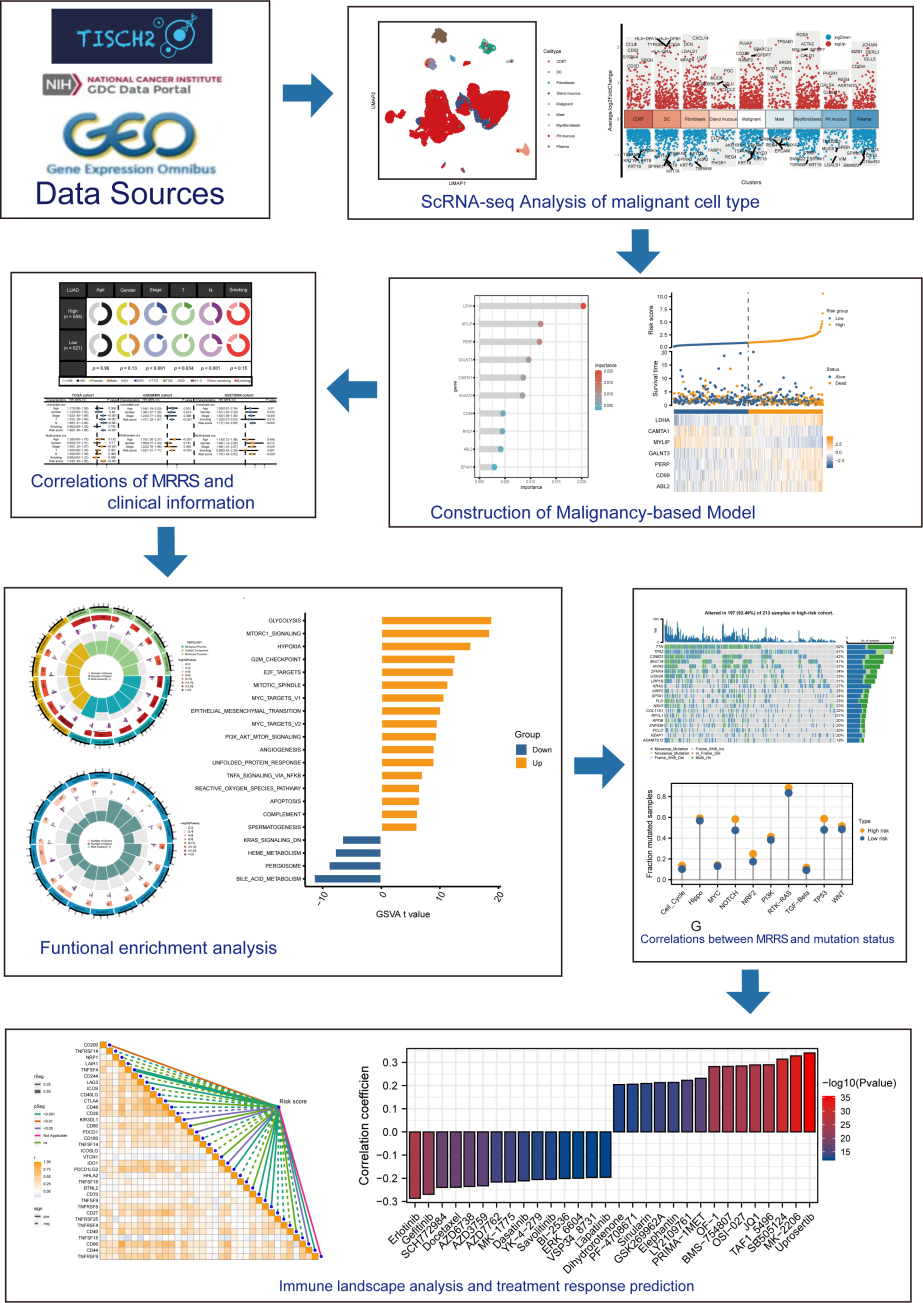
This study’s design and flowchart.

**Figure 2:**
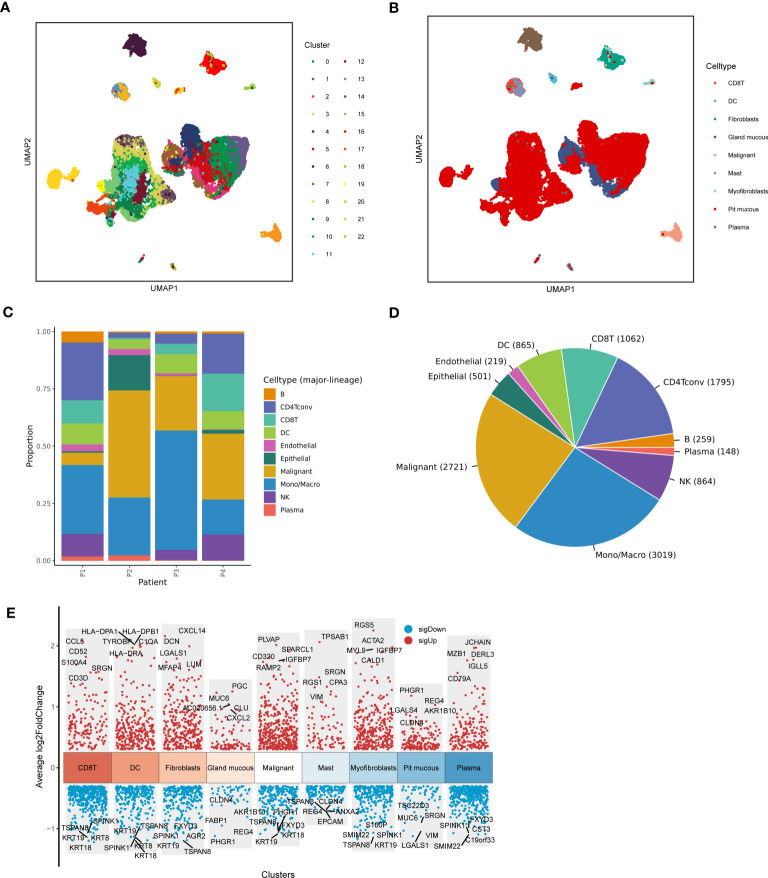
Sc-RNA seq analysis of LUAD. **(A)** The UMAP clustering map showed different clusters. **(B)** The UMAP clustering map showed different celltypes. **(C, D)** The proportion and construction of different celltypes. **(E)** Marker genes of different celltypes.

**Figure 1** depicted the flowchart for this research. ScRNA-seq data of 4 LUAD patients were downloaded from the GSE117570 dataset. After data processing, standardization, and data filtering, a total of 11,453 cells were obtained for subsequent analysis. After unsupervised clustering of all cells, 22 clusters were obtained (**Figure 2A**), which were visualized after dimensionality reduction by UMAP. According to the annotation of the TISCH database, we classified the cells into 9 cell types, including CD8 T cells, dendritic cells, fibroblasts, gland mucous cells, malignant cells, mast cells, myofibroblasts, pit mucous cells, and plasma cells (**Figures 2B–D**). The differentially expressed genes of all cell types were displayed in **Figure 2E**. Considering the key role of malignant cells in tumor tissue, we extracted the markers genes of malignant cell type for further study.

### 3.2 Construction of malignancy-based model

**Figure 3:**
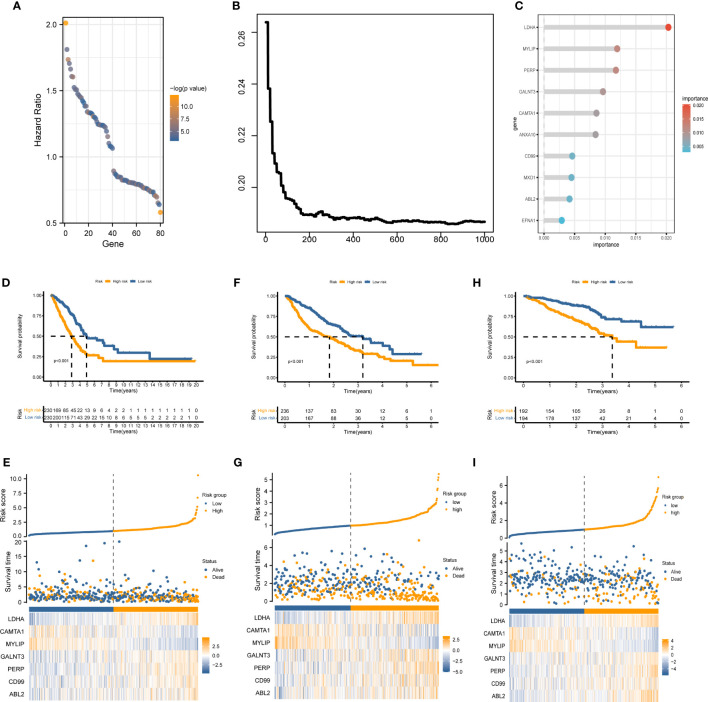
The establishment of MRRS and verification of its prognostic efficiency. **(A)** Univariate Cox regression analysis recognized 80 prognosis-associated genes. **(B)** Correlations between error rate and classification trees. **(C)** The relative importance of prognosis-associated genes. **(D)** The Kaplan-Meier method unveiled a significantly worse OS of the high-risk cohort compared to the low-risk cohort. **(E)** The illustrations of all patient’s survival conditions, risk variations, and MRRS distributions. **(F–I)** The outcomes of the GSE68465 and GSE72094 cohorts also validated MRRS’s prognostic value.

**Figure 4:**
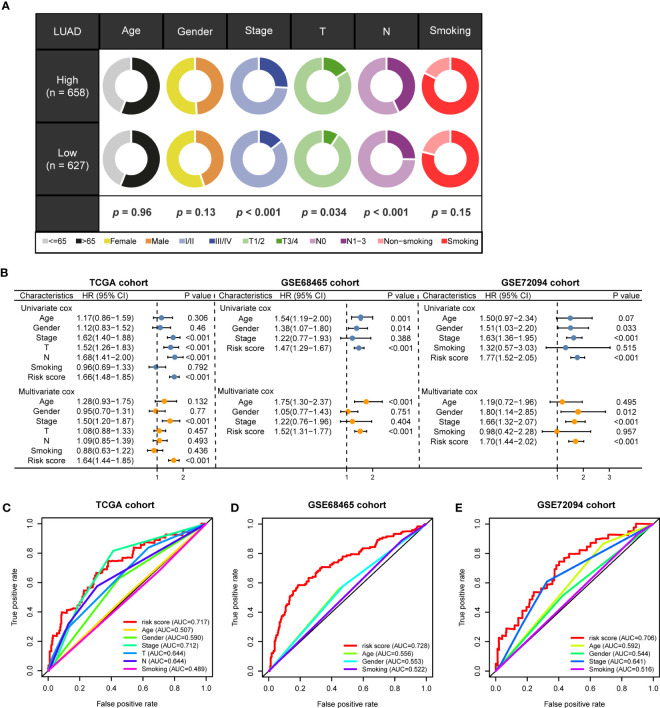
The prognostic value of MRRS and clinical variables. **(A)** Relationship between MRRS and clinical features. **(B)** Univariate and multivariate Cox regression analyses of MRRS and clinical features in TCGA, GSE68465, and GSE91061 cohorts. **(C–E)** The ROC method revealed the prognostic significance of MRRS in TCGA, GSE68465, and GSE72094 cohorts, respectively.

The marker genes of malignant cells were incorporated into the following study. In TCGA cohort, univariate Cox regression analysis recognized 80 prognosis-associated genes (**Figure 3A**). RSF analysis further identified 10 model-constructed candidates based on the minimal depth method (**Figures 3B, C**). Seven vital genes were ultimately chosen to form the MRRS using multivariate Cox regression, namely LDHA, CAMTA1, MYLIP, GALNT3, PERP, CD99, and ABL2. The formula was: $MRRS=\sum_{i=1}^{7}\left(Exp_{i}*\beta_{i}\right)$ (**Table 1**). Based on the median MRRS, the patients were separated into high-risk and low-risk. The OS of the high-risk group was significantly shorter than that of the low-risk group (**Figure 3D**). **Figure 3E** showed these patients’ MRRS distribution, survival status, and MRRS profile. Further analyses suggested that a higher MRRS was correlated with worse clinical stage and TN stages (**Figure 4A**). In addition, it was observed that MRRS and clinical variables were closely associated with OS in the univariate Cox regression. Further multivariate Cox analysis indicated that MRRS was an independent prognostic factor (**Figure 4B**). ROC analysis also confirmed the predictive efficacy of MRRS (AUC = 0.717, **Figure 4C**). The outcomes of the GSE68465 and GSE72094 cohorts also validated MRRS’s prognostic value (**Figures 3F–I**, **4**). These outcomes showed that MRRS was a highly reliable prognostic indicator.

**Table 1:** The prognostic significance of the 7-genes signature.

MRRS-related gene	Coef
LDHA	0.444007897
CAMTA1	-0.159218411
MYLIP	-0.337064301
GALNT3	0.128249103
PERP	0.111381641
CD99	0.15017571
ABL2	0.234266034

### 3.3 Functional enrichment analysis

**Figure 5:**
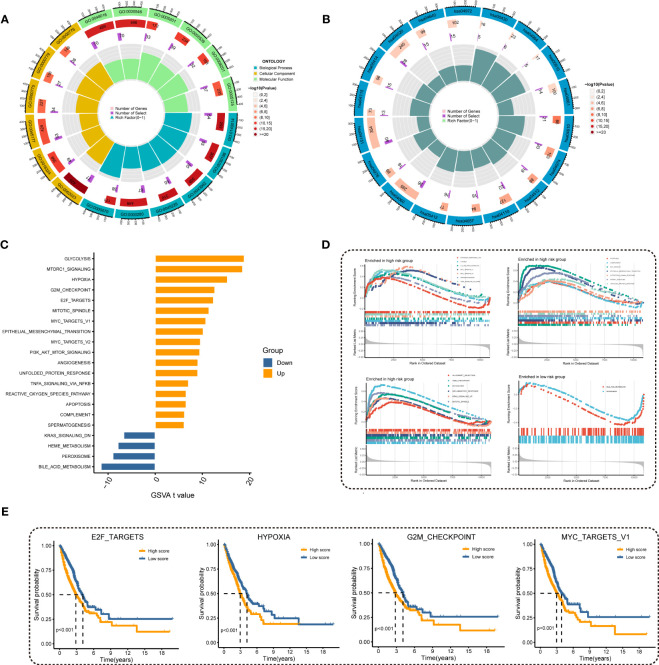
Investigation of underlying mechanism regarding MRRS. **(A)** GO enrichment analysis of MRRS. **(B)** KEGG pathway analysis of MRRS. **(C)** Determination of oncogenic hallmark pathways in terms of the MRRS risk cohorts utilizing GSVA. **(D)** The GSEA outcomes for the hallmark pathways between the high- and low-risk patients. **(E)** Kaplan-Meier curve uncovered the OS in overlapping hallmark pathways between GSVA and GSEA.

To investigate the underlying mechanism regarding MRRS, we performed GO and KEGG analyses and the results revealed that MRRS was related to receptor ligand activity, signaling receptor activator activity, extracellular matrix organization, myeloid leukocyte migration, cell cycle, chromosomal region, and mitotic nuclear division (**Figures 5A, B**). The above GO and KEGG items suggested that MRRS may be involved in oncogenic pathways, tumor mutations, and immune functions. Subsequently, 50 oncogenic hallmark pathways were included in GSVA, whose outcomes indicated that 17 hallmark pathways were significantly increased in high-risk patients while 4 pathways were decreased in low-risk patients (**Figure 5C**). GSEA confirmed that 20 were significantly upregulated in the high-risk cohort, while 2 were upregulated in the low-risk cohort (**Figure 5D**). The pathways obtained by intersection were analyzed by the Kaplan-Meier method, and different OS probabilities were observed for several well-known oncogenic pathways such as E2F_TARGETS, HYPOXIA, G2M_CHECKPOINT, and MYC_TARGETS_V1 (**Figure 5E**). In summary, MRRS participated in multiple biological functions, especially oncogenic pathways in LUAD.

### 3.4 Somatic mutation analysis

**Figure 6:**
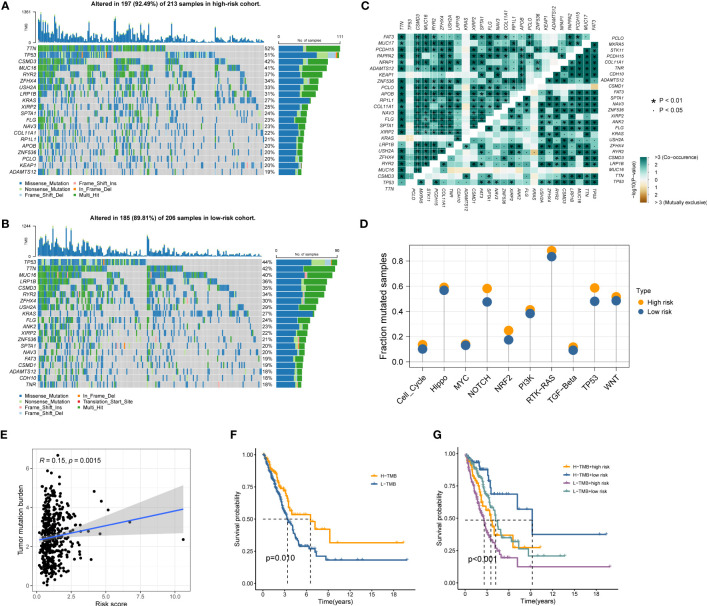
Genetic mutations landscape in terms of the MRRS risk cohorts. **(A, B)** Waterfall plots of genetic mutations in high- and low-risk groups, respectively. **(C)** The co-occurring or exclusive mutations across the top 25 mutated genes between the two cohorts. **(D)** The results of mutation enrichment of remarkable oncogenic pathways. **(E)** The relationship of MRRS and TMB. **(F, G)** Kaplan-Meier curve revealed the OS in distinct TMB and MRRS groups.

Gene mutations landscape between the high- and low-risk groups were shown in waterfall plots (**Figures 6A, B**). The genes with the highest mutation frequencies in the high-risk cohort were TTN, TP53, CSMD3, MUC16, and RYR2, while those in the low-risk cohort were TP53, TTN, MUC16, LRP1B, and CSMD3. Furthermore, the co-occurring or exclusive mutations across the top 25 mutated genes between the two cohorts were also exhibited, with no significant differences observed (**Figure 6C**). The mutation enrichment of known oncogenic pathways showed no significant difference between the high- and low-risk teams (**Figure 6D**). Further analysis also confirmed the positive correlation between TMB and MRRS, and higher TMB had a better OS (**Figures 6E, F**). Survival analysis suggested that low TMB and high MRRS had the worst prognoses (**Figure 6G**). In conclusion, comprehensive analyses disclosed the mutation variations between high- and low-risk cohorts, and multiple remarkable genes and pathways showed significant mutation abnormalities between the cohorts.

### 3.5 Immune landscape analysis and treatment response prediction

**Figure 7:**
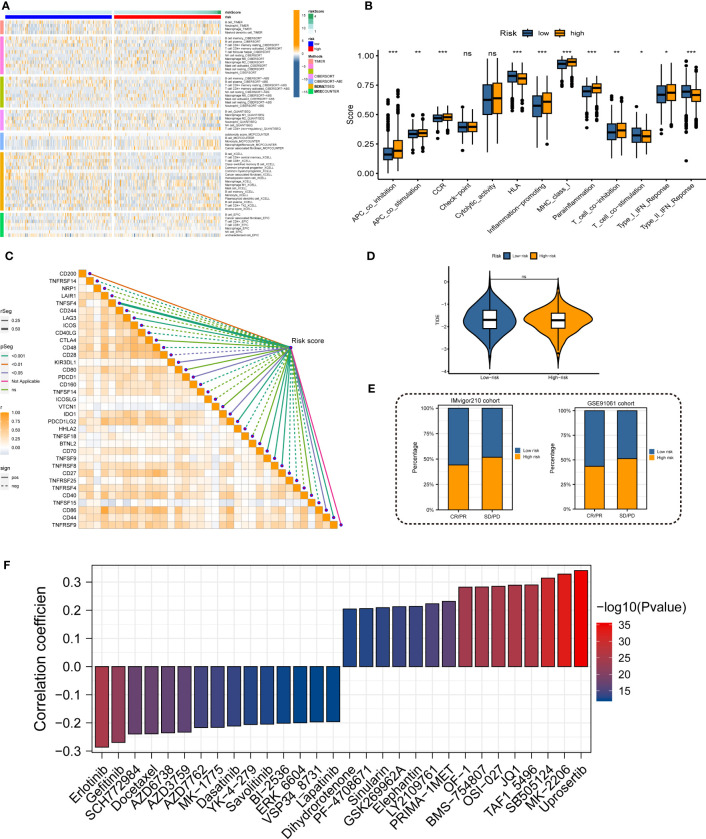
Immune landscape and treatment response prediction. **(A)** Estimation of immune cell infiltration in high- and low-risk teams. **(B)** Explorations of immunological responses in terms of the MRRS risk groups. **(C)** Correlations between MRRS and immune checkpoints. **(D)** TIDE algorithm identified the difference in immunotherapy response between high- and low-risk groups. **(E)** The prediction of immunotherapy response using IMvigor210 and GSE72094 cohorts. **(F)** The prediction of chemotherapy response of LUAD patients with different MRRSs. *p< 0.05, **p< 0.01, ***p< 0.001, ns, not significant.

Immune landscape analysis revealed higher abundances of T cells, B cells, and NK cells in the low-risk group compared to the high-risk group (**Figure 7A**). Most anti-tumor immune functions such as HLA function and T cell co-stimulation were relatively decreased in the high-risk team (**Figure 7B**). Besides, we also found higher expressions of immunosuppressive receptors and inhibitory ligands (PDCD1, PDCD1LG2, and LAG3) in high-risk patients (**Figure 7C**). Meanwhile, the TIDE algorithm identified no significant difference in immunotherapy response between the high- and low-risk groups (**Figure 7D**). The predicted outcomes of the IMvigor210 cohort and GSE91061 cohort also supported the above conclusion (**Figure 7E**). Considering the poor response to immunotherapy in LUAD, the chemotherapy response of LUAD patients with different MRRSs was assessed by the “oncoPredict” R package. Our findings indicated that high-risk patients had significantly lower IC50 values in several chemotherapy molecules including Erlotinib, Gefitinib, SCH772984, and Docetaxel (**Figure 7F**). Overall, the immune landscape analyses demonstrated that MRRS was associated with different immune responses, and chemotherapy may be more effective than immunotherapy for the high-risk patients.

### 3.6 Expression level of MRRS-constructed genes in LUAD cells

**Figure 8:**
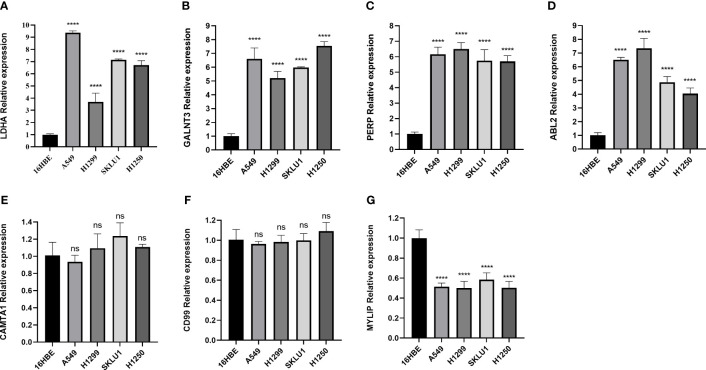
The qRT-PCR result for the MRRS-constructed genes. **(A)** LDHA. **(B)** GALNT3. **(C)** PERP, **(D)** ABL2, **(E)** CAMTA1, **(F)** CD99, **(G)** MYLIP.

In addition, we validated the expression levels of MRRS-constructed genes (LDHA, CAMTA1, MYLIP, GALNT3, PERP, CD99, and ABL2) between the LUAD cells (A549, H1299, SKLU1, and H1250) and normal bronchial epithelial cells (16HBE) using qRT-PCR. As shown in **Figure 8**, the expression of LDHA, GALNT3, PERP, and ABL2 was significantly upregulated in LUAD cell lines, while MYLIP was significantly upregulated in 16HBE cells. However, the expression of CAMTA1 and CD99 showed no significant difference between the LUAD cells and normal bronchial epithelial cells. Overall, the results of qRT-PCR were consistent with bioinformatics analysis.

## 4 Discussion

Patients with lung cancer, with the highest fatality rate in the world, had a dismal prognosis, with 5-year survival rates of less than 20% (3). Patients with advanced LUAD now have new hope thanks to the development and application of immunotherapy and targeted therapies medications in recent years (15). However, due to molecular variances, patients with the same pathological type and clinical stage may have varying prognoses. Therefore, to predict the prognosis of LUAD patients, further molecular indicators must be investigated. Increasing bioinformatic articles get published recent years and achieved excellent efficacy (6, 16–19). The scRNA-seq has good application potential in disease research since it can obtain gene expression maps at the level of a single cell and identify heterogeneous tissue samples in groups (20–22). In our study, we amalgamated data from TCGA, GSE68465, and GSE72094 cohorts to acquire MRRS. ROC analysis verified the advantageous predictive efficacy of MRRS with an AUC of 0.717. Multiple datasets were utilized to investigate the biological functions of MRRS, which augmented the reliability of the results.

Furthermore, GO and KEGG analyses demonstrated that MRRS may be correlated with chromosomal region, mitotic nuclear division, extracellular matrix organization, myeloid leukocyte migration, cell cycle, and signaling receptor activator activity. The findings of GSVA and GSEA implied that MRRS may modify the tumor’s biological behavior by participating in multiple oncogenic hallmark pathways. Signal pathways including E2F TARGETS, HYPOXIA, G2M CHECKPOINT, and MYC TARGETS V1 were found to be strongly correlated with patient prognosis by Kaplan-Meier analysis. It has been reported that upregulation of the E2F signaling promoted the proliferation and progression of LUAD (23). The MYC pathway’s aberrant activation can contribute to the progression of LUAD and its metastasis (24, 25). As a result, by regulating certain oncogenic pathways, MRRS may have an impact on a patient’s prognosis.

The genetic mutations between the high- and low-risk groups were further investigated. TTN, TP53, CSMD3, MUC16, and RYR2 were the top five genes for mutation frequency in the high-risk team, while TP53, TTN, MUC16, LRP1B, and CSMD3 were the top five genes in the low-risk team. LRP1B was reported as a tumor suppressor gene in non-small-cell lung cancer (26). A recent study showed that patients with cancer who had LRP1B mutations had improved clinical outcomes when receiving immune checkpoint inhibitors (27). Additionally, we discovered that MRRS and TMB had a positive correlation and that their combination can more accurately predict the prognosis of the patient. TMB has emerged as a valuable biomarker for evaluating the efficacy of immunotherapy in LUAD because it represents a mutagenesis process triggered by intracellular and environmental factors (28, 29). Our research identified mutation variations in the MRRS risk cohorts. MRRS may take part in a variety of aberrant mutations involving oncologic genes and pathways to modulate the growth and progression of LUAD.

Immune landscape analysis revealed that the high-risk group had substantially more immune cells and immune functions. Consequently, an additional investigation discovered that the high-risk patients had a high expression of immunosuppressive receptors and inhibitory ligands (PDCD1, PDCD1LG2, and LAG3). Immune checkpoint inhibitors, as we all know, have recently significantly improved the prognosis for patients with lung cancer (30), but they are only beneficial in a small subset of patients with LUAD. The majority of LUAD patients were still receiving targeted therapy and chemotherapy as their basic treatments. The results revealed that the high-risk group responded better to erlotinib, gefitinib, SCH772984, and docetaxel. Overall, the immune landscape analysis showed that MRRS was involved in many immune responses and that, for high-risk patients, chemotherapy may be more efficient than immunotherapy.

In our research, the LDHA, CAMTA1, MYLIP, GALNT3, PERP, CD99, and ABL2 genes are vital ingredients of the MRRS. Among them, LDHA showed a substantial correlation with the prognosis of LUAD, which is consistent with earlier researches (31, 32). According to a recent study, phosphorylation and activation of LDHA can promote tumor invasion and metastasis (33). By regulation of LncRNA SGMS1-AS1 and miR-106a-5p38, MYLIP, a potential tumor suppressor gene in LUAD, may prevent the proliferation, invasion, and migration of LUAD cells (34). GALNT3 was developed to prevent lung cancer by preventing self-renewal and the development of a favorable tumor microenvironment (35). Previous studies showed that the knockdown of ABL2 dramatically reduced the brain metastases of LUAD cells (36, 37). According to the above findings, possible treatments targeting these essential genes may exist in the future.

Our study still had significant shortcomings, though. Since this research was retrospective, additional treatment and relapse records as well as prospective clinical investigations are needed to verify our findings. These essential genes will require additional experimental validation, and additional *in vivo* or *in vitro* experiments are needed to investigate the specific mechanisms of the genes.

## 5 Conclusion

In conclusion, we developed a novel malignancy-related signature, termed MRRS, which demonstrated a strong predictive capacity for OS in LUAD patients. Our findings shed light on the underlying mechanisms of LUAD progression and suggest that MRRS may serve as a promising prognostic and therapeutic marker for LUAD patients.

## Data availability statement

The original contributions presented in the study are included in the article/**Supplementary Material**. Further inquiries can be directed to the corresponding author.

## Author contributions

JHZ designed and started the research. MW, ZW, and JY analyzed the data and drafted the manuscript. JZ, JK, CZ, and XZ revised the manuscript. YM, QG, DL, JT, and TZ collected the data. All authors contributed to the article and approved the submitted version.

## Footnotes

1
http://tisch.comp-genomics.org/

2
https://portal.gdc.cancer.gov/

3
https://www.ncbi.nlm.nih.gov/geo/

4
http://tide.dfci.harvard.edu/

5
https://github.com/No-Potato/LUAD-MRRS
